# Case report: Successful immunomodulators combined with electromagnetic field therapy in a patient with methazolamide-induced Steven Johnson syndrome/toxic epidermal necrolysis overlap

**DOI:** 10.3389/fmed.2023.1192920

**Published:** 2023-05-25

**Authors:** Naiju Zhang, Tianjiao Su, Jingwen Yan, Mei Zhang, Shousong Zhao, Chuanmiao Liu, Tianping Chen

**Affiliations:** ^1^Department of Pharmacy, First Affiliated Hospital of Bengbu Medical College, Institute of Emergency and Critical Care Medicine, Anhui Engineering Technology Research Center of Biochemical Pharmaceutical, Bengbu, Anhui, China; ^2^Key Laboratory of Immunology in Chronic Diseases, Department of Infectious Diseases, National Clinical Research Center for Infectious Diseases, First Affiliated Hospital of Bengbu Medical College, Bengbu, Anhui, China; ^3^Department of Cardiology, First Affiliated Hospital of Bengbu Medical College, Bengbu, Anhui, China

**Keywords:** methazolamide, Steven Johnson syndrome, toxic epidermal necrolysis, adverse drug reaction, electromagnetic field therapy, case report

## Abstract

Methazolamide is used to treat patients with glaucoma. However, as a sulfonamide derivative, methazolamide shares the same adverse reaction profile as other sulfa-based medications. Stevens–Johnson syndrome (SJS) and toxic epidermal necrolysis (TEN) are rare delayed-type hypersensitivity cutaneous reactions with high morbidity and mortality. Here, we report a severe SJS/TEN overlap syndrome in an 85-year-old Chinese male patient who received methazolamide 25 mg twice daily for his left eye glaucoma. The causal relationship between SJS/TEN and methazolamide was categorized as “highly likely” on the algorithm for assessing drug causality for epidermal necrolysis. In addition to the treatments with methylprednisolone and immunoglobulin, we used a special electromagnetic spectrum therapeutic apparatus to provide skin wound care. The patient had a thoroughly satisfying recovery. This is the first case report to use electromagnetic field therapy in a patient with SJS/TEN. We share our experience here and suggest that electromagnetic field therapy can provide advanced skin wound care and facilitate the recovery of SJS/TEN.

## Introduction

Methazolamide is a carbonic anhydrase inhibitor used in patients with glaucoma (1). It can reduce aqueous humor formation to decrease intraocular pressure. However, as a sulfonamide drug, methazolamide may cause severe adverse reactions. Stevens–Johnson syndrome (SJS) and toxic epidermal necrolysis (TEN) are delayed-type hypersensitivity cutaneous reactions (2). The difference between SJS and TEN is their skin area involvement, with <10, 10–30, and >30% of body surface area for the diagnosis of SJS, SJS/TEN overlap, and TEN, respectively. In multiple locations, patients with SJS or TEN can have epidermal loss and mucositis. Severe cases can even have systemic involvements and multiple organ dysfunctions, with the mortality rates reaching 1–5% and 25–30% for SJS and TEN, respectively (3). The pathogenesis of SJS/TEN disease is still under investigation. The massive keratinocyte death and the Fas-Fas ligand binding-mediated cytotoxic T cell and caspase 8 activation were considered to participate in the development of the SJS/TEN (4, 5).

Electromagnetic field therapy has recently been studied as a non-invasive and safe method to treat musculoskeletal disorders. The microcurrent produced under the magnetic field could stimulate living cells to reproduce and repair injured tissues. In addition, to provide pain relief (6), animal studies and clinical trials have shown the benefits of electromagnetic field therapy during the management of dermatitis, arthritis, and bone fracture (7–9).

Here, we report a severe rare case of SJS/TEN overlap syndrome caused by methazolamide, which was successfully treated by immunomodulatory and electromagnetic field therapies. Informed consent was obtained from the patient to publish this case report.

## Case report

An 85-year-old Chinese male patient was admitted to the department of infectious diseases of the first affiliated hospital of Bengbu medical college, Bengbu, China, with pruritic rashes and fever on October 17, 2019. His medical history included cerebral infarction, left-eye glaucoma, and right-eye blindness. The patient denied a history of drug or food allergies. In contrast, his daughter had a history of labial ulceration after taking sulfanilamide tablets, which improved within 2 days after discontinuing the medication.

Twelve days before hospital admission, the patient was prescribed methazolamide (25 mg tablets, twice daily for 10 days) to treat glaucoma. One day after taking the medication, the patient developed swollen red eyes. He went to a local hospital and was diagnosed with corneal injury and keratitis. He received antibiotic treatment with levofloxacin eye drops and additional medications (carteolol eye drops, furan thiamine tablets, and pilocarpine eye drops) for his glaucoma. Six days before admission, the patient started to have a fever, with the highest temperature reaching 39.9°C. Three days later, he noticed itching pale red macules with blister formations, starting from the abdomen, genitals, hands, and soles but quickly spreading all over his body. He revisited the local hospital. A dermatologist was consulted, which considered severe erythema multiforme. Blood tests showed a white blood cell count of 6.3 × 10^9^/L (normal range 3.5–9.5 × 10^9^/L), neutrophil ratio 84.6% (normal range 40–70%), and procalcitonin 0.1 ng/mL (normal range <0.1 ng/mL). In addition, chest computed tomography imaging revealed bilateral pneumonia. The patient received cefuroxime with supportive and symptomatic care. However, his fever and rashes failed to improve.

During the admission to our hospital, the patient was awake and alert, with the vital signs, temperature 38.7°C, pulse 80 times/min, respiratory rate 18 times/min, and blood pressure 107/68 mmHg. His eyes were red, swollen, and filled with fluid. He had glaucoma in his left eye, with a pupil diameter of 3 mm. He was sensitive to light reflexes and had a visual acuity of 0.2. His right eye was blind. The patient had scattered itching erythematous macules coalescing into papules and blisters all over the body (Supplementary Figure 1), with no epidermal detachment. He also had ulcerations in the oral mucosa and right foot. Nikolsky's sign was positive. Otherwise, his pulmonary, cardiac, abdominal, and neurological examinations were unremarkable. Laboratory tests showed the white blood cells count 6.5 × 10^9^/L, neutrophil percentage 79.4%, C-reactive protein 30.6 mg/L (normal range 0–10 mg/L), and procalcitonin 0.1 ng/mL. Based on his clinical presentations and the algorithm for the assessment of drug causality for epidermal necrolysis (ALDEN) (10), we made the diagnosis of SJS induced by methazolamide (a total score of 8, Table 1). We discontinued all his previous medications and treated him with methylprednisolone 40 mg twice daily and intravenous immunoglobulin 30 g daily. In addition, albumin was administered to alleviate exudation and improve hypoproteinemia.

**Table 1:** Algorithm of drug causality for epidermal necrolysis based on the ALDEN criteria (10).

**Recommended ALDEN criteria**		**Present case report**	
**Criterion**	**Values**	**Rules to apply**	**Patient's evidence**	**Patient's score**
Delay from initial drug component intake to the onset of reaction (index day)	Suggestive +3	From 5 to 28 days	11 days	+3
Drug present in the body on the index day	Definite 0	Drug continued up to index day or stopped at a time point less than five times the elimination half-life before the index day.	The plasma elimination half-life of methazolamide was 14 h. One day post-methazolamide withdrawal, the patient exhibited red and swollen eyes.	0
Prechallenge/rechallenge	Positive specific for disease or drug: 2	SJS/TEN after the use of a similar drug or other reaction with the same drug	The patient's daughter had a history of labial ulceration after taking sulfanilamide tablets, which improved 2 days after stopping the medication	2
Dechallenge	Neutral 0	Drug stopped (or unknown)		0
Type of drug (notoriety)	Strongly associated 3	Drug on the “high-risk” list according to previous case-control studies	Methazolamide belongs to the sulfonamides family	3
Final score				8

**Figure 1:**
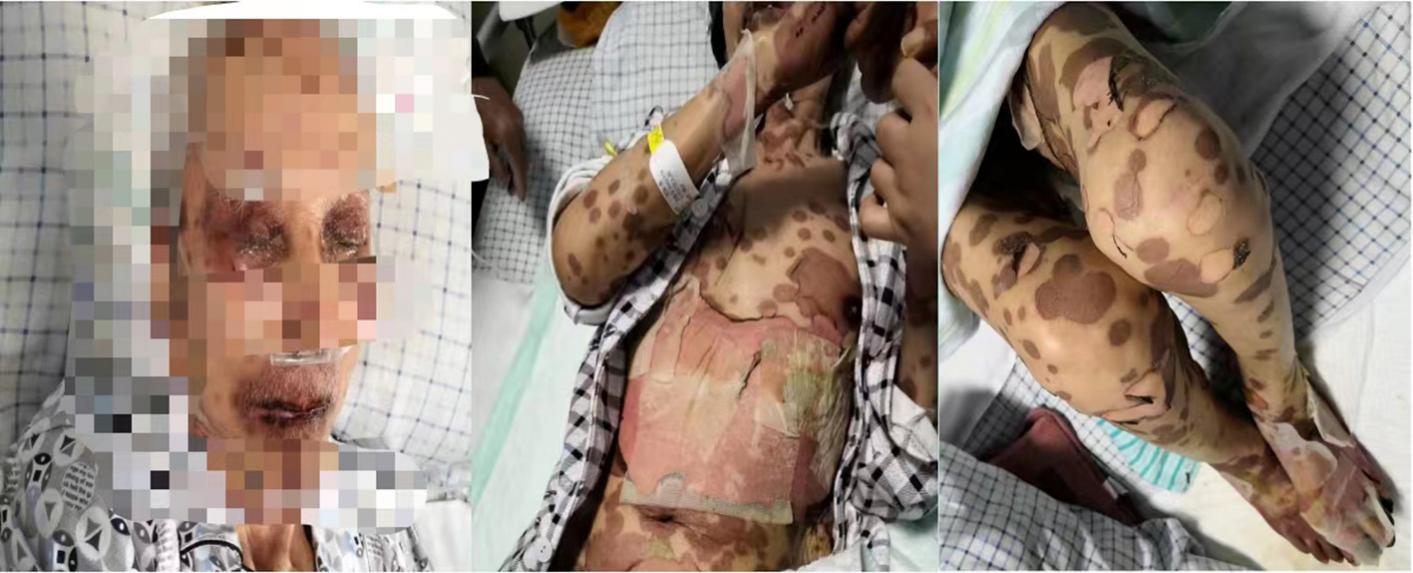
Skin conditions on the seventh day of hospitalization.

**Figure 2:**
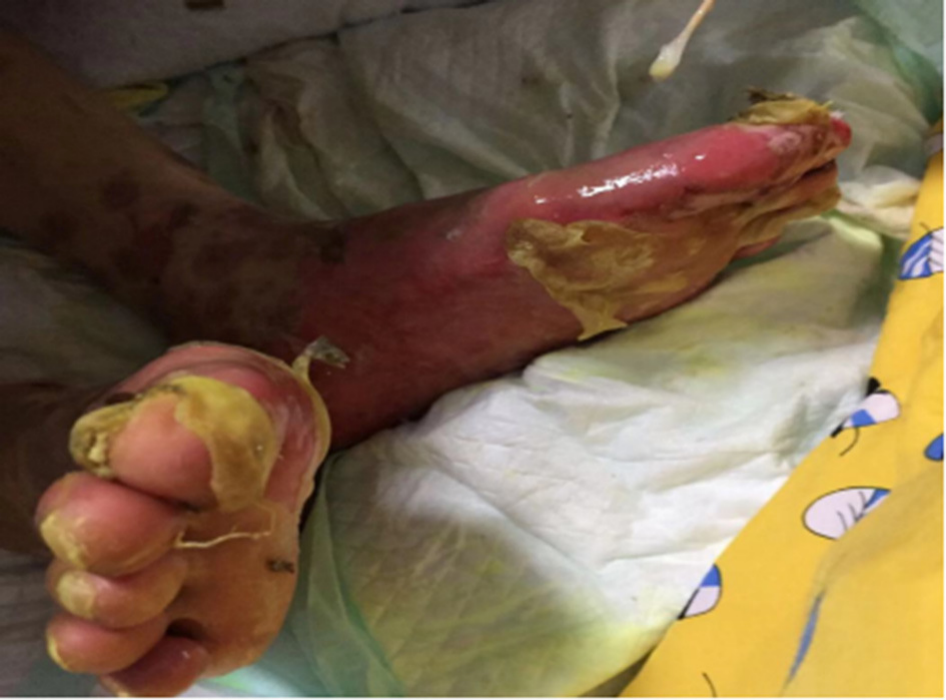
Skin conditions on the feet on the 13th day of hospitalization.

On the 7th day of hospitalization, the patient had rashes all over the body, accompanied by local ulceration, with 15–20% body surface area having epidermis shedding and significant exudations (Figure 1). On the 13th day of hospitalization, the patient had extensive blisters on both hands and anterior chest wall. The rash in his back fused into a sheet, followed by extensive denudation and erosion. There were also bilateral plantar skin peeling, subcutaneous tissue flushing, copious exudations, and epidermal detachment ≥ 80% of body surface area (Figure 2). His final diagnosis was the SJS/TEN overlap syndrome due to methazolamide.

After treatments with methylprednisolone and immunoglobulin, the tiny blisters gradually shrank as the underlying skin recovered. For large blisters, we used needle aspirations to remove the fluids. Skin lesions were covered by Vaseline gauze strips and sterile gauze soaked with ethacridine. Alginate was applied in his chest, right upper arm skin, and feet to stop the bleeding during skin exfoliation. For foot ulcers, we used iodophor as a disinfectant while covering the ulcers with a rivanol oil gauze.

On the 15th day of hospitalization, we applied electromagnetic field therapy to facilitate his skin wound care, in addition to the previously mentioned treatments. A special electromagnetic spectrum therapeutic apparatus (Supplementary Figure 2) (Chongqing Xinyi Medical Equipment Co., LTD, license: Chongqing food and drug regulatory machinery production permit, China) was placed on the overbed cradle with an irradiation distance of 20–40 cm and the irradiation time of 10–15 min, two or three times a day. Both the irradiation distance and time were adjusted according to the local skin temperature based on the manufacturer's instructions. Electromagnetic field therapy lasted for 9 days.

Approximately 20 days after hospitalization, the patient's temperature was 39°C, white blood cells count 8.2 × 10^9^/L, neutrophil percentage 94.1%, C-reactive protein 40.6 mg/L, and procalcitonin 0.1 ng/mL. His blood cultures were positive for *Staphylococcus hominis, Corynebacterium striatum*, and *Escherichia coli*. The patients received linezolid 0.6 g twice daily, vancomycin 0.5 g twice daily, and successively meropenem 1 g every 8 h. The blood beta-D-glucan fungal antigen test was 155.9 pg/ml (normal range <70 pg/ml). Oral and genital secretion smears revealed the presence of fungal spores. Nystatin ointment was applied to the white patches in the mouth. Naftifine and ketoconazole creams were applied on the white erosions in the scrotum, prepuce, and glans.

**Figure 3:**
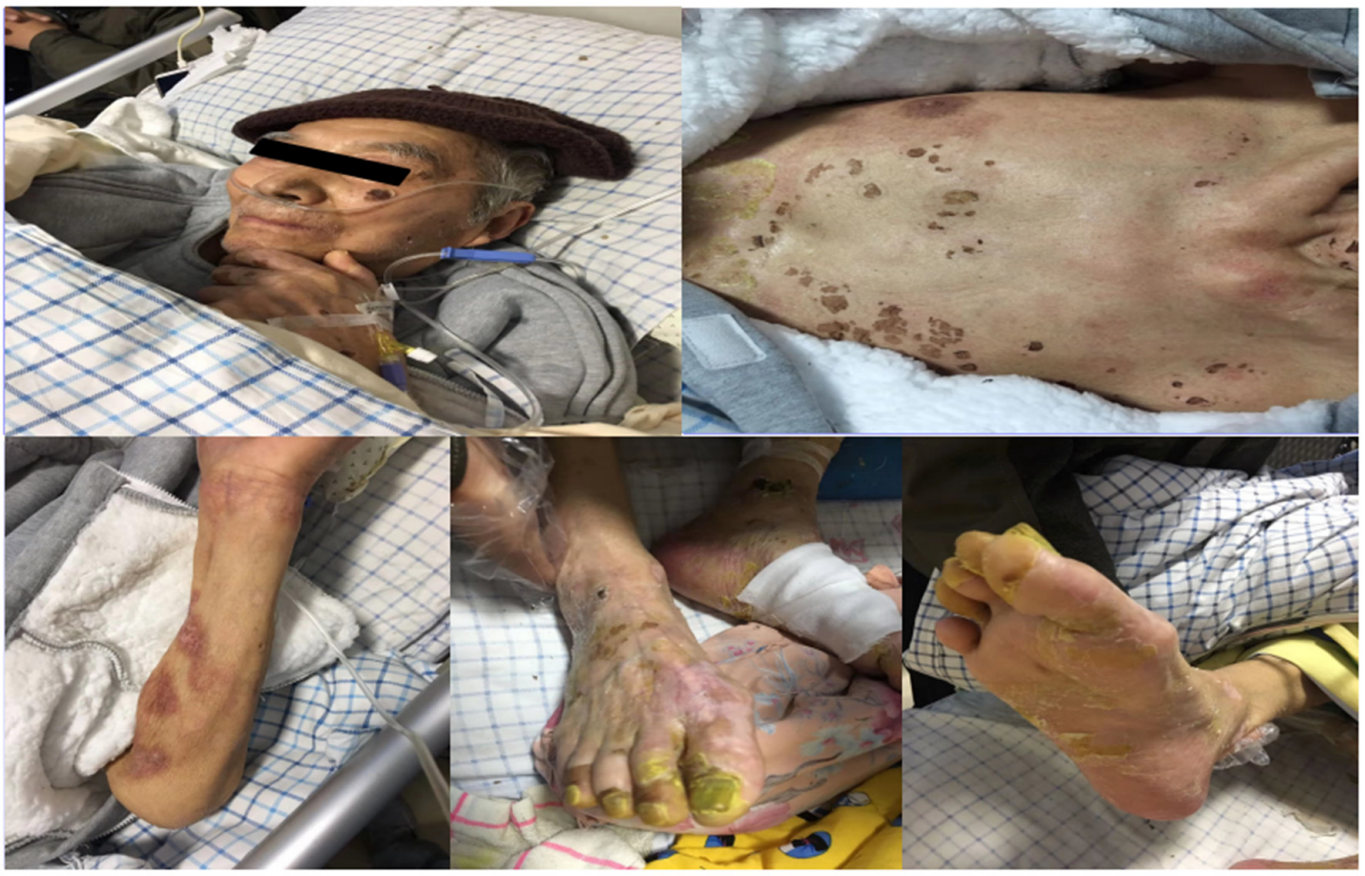
Skin conditions on the 39th day of hospitalization.

On the 29th day of hospitalization, scabs formed around the eyes and lips. The blisters began to crust, and the ulcers started to heal. The exudations in the front chest, back, and feet had reduced (Supplementary Figure 3). On the 39th day of hospitalization, the patient's body temperature and inflammatory indicators returned to normal. The fresh skin was visible all over the body (Figure 3). The skin crusts around both eyes fell off, and there was no apparent redness or swelling. He had glaucoma in his left eye, with a pupil diameter of 3 mm. He was sensitive to light reflexes and had a visual acuity of 0.2. His right eye was blind. The patient could also take food orally. After discontinuation of all drugs, his condition remained stable. The patient was discharged after a prolonged hospital stay of 42 days. The patient had no significant long-term sequelae at the clinical follow-up visit 3 years later.

The patient provided this perspective: “At present, skin and mucous membranes are fresh and smooth all over my body. The left leg was injured in a fall three years ago and now has a little pain and needs crutches to walk, my vision is still the same as before, only my left eye has vision, my memory is clear, and my appetite, stool and urine are normal, I've got another 20 years to live, that disease not only no sequelae, but also many years of stomach disease to cure, before I could not ate a lot of food, anything is fine now. Thank you very much.” Informed consent was obtained from the patient to publish this case report.

## Discussion

In this case report, we describe an 85-year-old Chinese man diagnosed with glaucoma admitted to our hospital with a severe drug rash. Twelve days prior to admission, the patient was prescribed methazolamide.

The algorithm for the assessment of drug causality for epidermal necrolysis (ALDEN) (10) score is widely recognized as one of the most reliable tools to identify the culprit drugs for SJS and TEN (3). The ALDEN score varies between −12 and 10, with a score ≥6, 4–5, 2–3, 0–1, and <0 corresponding to highly likely, probably, possible, unlikely, and very unlikely cause, respectively, for a medication. The final ALDEN score for this patient was 8 (Table 1). Methazolamide was considered a highly likely cause of the SJS/TEN overlap syndrome in this patient. The patient had a negative test for HLA-B^*^58:01 AA, which is a marker for SJS/TEN caused by allopurinol. Unfortunately, our hospital did not have a program for testing HLA-B59:01. We could not find any hospitals or companies that carried out HLA-B59:01 testing programs, which is considered a marker for SJS/TEN caused by methazolamide in the Han Chinese population (11).

SJS/TEN is a delayed-type hypersensitivity life-threatening cutaneous reaction to medication with extensive epithelial keratinocyte apoptosis and necrosis (2). The pathogenesis of drug-induced SJS and TEN involves T lymphocyte activation and various cytokine and chemokine productions to trigger widespread keratinocyte death. These finally result in severe skin lesions in SJS/TEN (12). Studies have confirmed that T cells had highly activated and expanded clones in SJS/TEN patients but a low T-cell receptor repertoire diversity, which could be associated with the clinical severity of the disease (13). Recent genetic studies confirmed the association of HLA-B^*^59:01 with methazolamide-induced SJS/TEN. The HLA-B^*^55:02 was reported as a novel risk allele for SJS/TEN in the Han population. In addition, the rs41562914(A)-rs12697944(A) haplotype was also proposed as a genetic marker for methazolamide-induced SJS/TEN with a specificity of 96% and sensitivity of 89% (14).

It should be noted that severe infection and sepsis are still the leading causes of death in SJS/TEN patients. Clinicians need to keep alert for early signs of sepsis or invasive infection. A delayed diagnosis without prompt intervention may lead to high mortality. Once sepsis or invasive infection is suspected, clinicians should quickly initiate appropriate antibiotics to cover the potential multidrug-resistant pathogens in these life-threatening situations (15). The patient's severe infection control was related to the timely use of broad-spectrum antibiotics.

Physicians should ask patients and their family members about previous sulfonamide drug allergies before prescribing methazolamide. If there is a history of sulfonamide drug allergy, medications containing sulfonamides or other drugs with a similar structure should be avoided. In addition, an HLA-B59:01, HLA-B^*^55:02, or methazolamide skin allergy test can be conducted before prescribing methazolamide to suspected patients. The screening of the rs41562914(A)-rs12697944(A) haplotype might avoid the occurrence of methazolamide-induced SJS/TEN in Han Chinese (14). During the methazolamide treatment, the patient body temperature, skin, and mucous membrane should be carefully monitored to allow the early diagnosis of SJS/TEN and prompt discontinuation of the methazolamide therapy. Once SJS/TEN is diagnosed, immunomodulatory therapy should be initiated. In addition, sophisticated skin wound care should be provided.

We used electromagnetic field therapy for advanced skin wound care in our patient. The electromagnetic field had wavelengths between 2 and 25 μm. The special electromagnetic spectrum therapeutic apparatus works by generating heat when powered on. The heat is transferred to the element plate through a mica insulation board, generating comprehensive electromagnetic waves of different wavelengths and energies. These waves resonate with responsive elements in the human body when irradiating subcutaneous tissue, achieving the effect of electromagnetic therapy. This therapy is suitable for auxiliary relief of conditions such as arthritis, lumbar disc herniation, rheumatic bone disease, and shoulder periarthritis. The produced magnetic field and warming effects have anti-inflammatory properties, which can reduce swelling, facilitate pain relief, decrease exudation, remove stasis, and promote blood circulation, metabolism, and epithelial growth to accelerate wound healing (16). Electromagnetic field therapy could be a treatment option to accelerate the skin wound healing process in patients with drug-induced SJS/TEN.

## Conclusion

Methazolamide-induced SJS/TEN is a life-threatening disease that requires prompt diagnosis and management, especially in elderly patients with a history of allergic reactions to sulfonamide. Electromagnetic field therapy can provide advanced skin wound care and facilitate the recovery of SJS/TEN.

## Data availability statement

The original contributions presented in the study are included in the article/Supplementary material, further inquiries can be directed to the corresponding authors.

## Ethics statement

The studies involving human participants were reviewed and approved by the Ethics Committee of the First Affiliated Hospital of the Bengbu Medical College (2020KY072). The patients/participants provided their written informed consent to participate in this study. Written informed consent was obtained from the individual(s) for the publication of any potentially identifiable images or data included in this article.

## Author contributions

NZ was responsible for data collection and drafting the manuscript. TS, JY, MZ, and SZ participated in the critical care of the patient. TC and CL designed the study, participated in drafting the manuscript, critically revised it for important intellectual content, and approved the final version for submission. All authors contributed to the article and approved the submitted version.
