# Supplementary material for: Case report: Successful immunomodulators combined with electromagnetic field therapy in a patient with methazolamide-induced Steven Johnson syndrome/toxic epidermal necrolysis overlap
Source: Front Med (Lausanne). 2023 May 25;10:1192920. doi: 10.3389/fmed.2023.1192920 (PMC10248229; doi:10.3389/fmed.2023.1192920)
Supplement: Supplementary file 1 [file Data_Sheet_1.PDF]

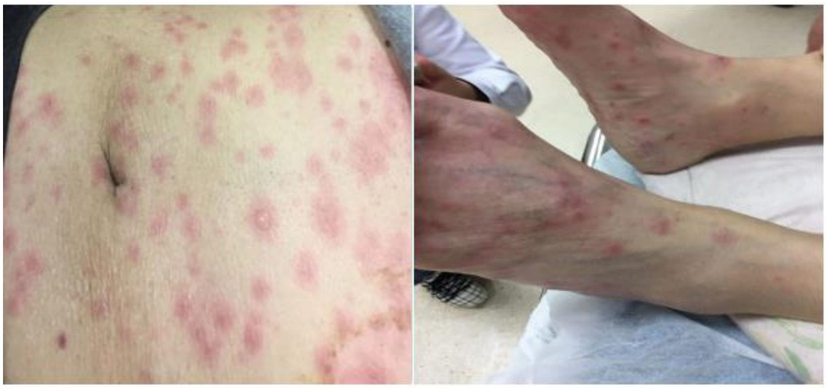

Supplementary fig.1. Skin conditions on chest, abdomen and feet at the hospital admission

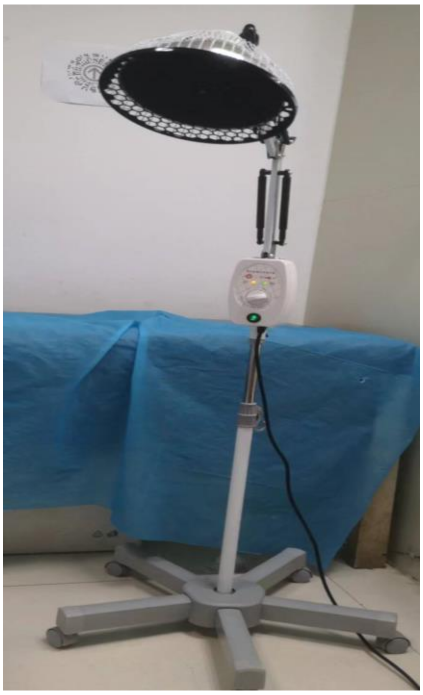

Supplementary fig.2. A special electromagnetic spectrum therapeutic apparatus

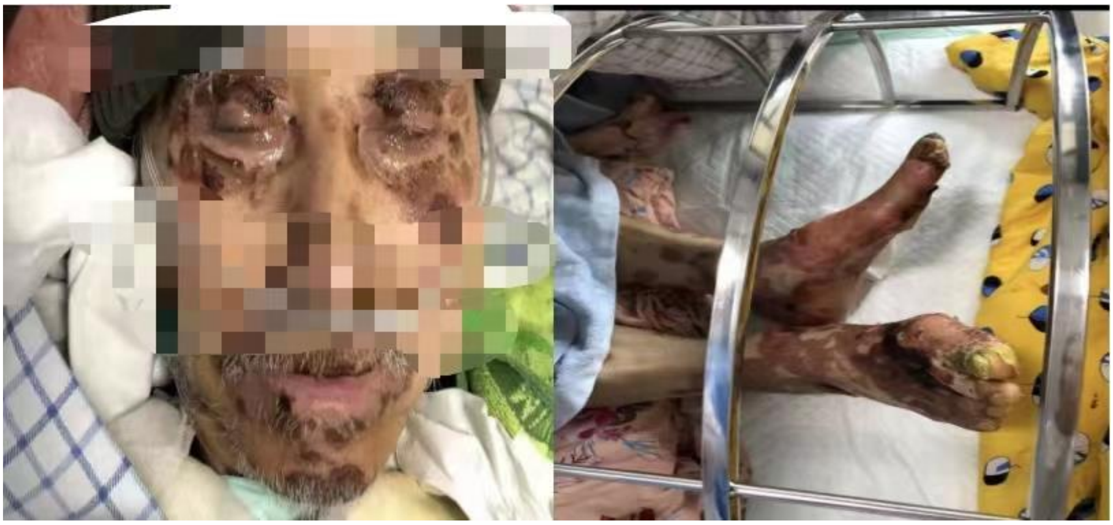

Supplementary fig. 3 Skin conditions on the feet on the 29th day of hospitalization
